# Phospholipid Transfer Protein (PLTP) in Cholesterol Handling: Implications for Mitochondrial Lipid Homeostasis in Human iPSC-Derived Cardiomyocytes

**DOI:** 10.3390/ijms27083617

**Published:** 2026-04-18

**Authors:** Dhienda C. Shahannaz, Tadahisa Sugiura

**Affiliations:** 1 Digestive Disease & Surgery Institute, Cleveland Clinic, Cleveland, OH 44195, USA; dhiendaladdynasrul@gmail.com; 2 Department of Cardiothoracic and Vascular Surgery, Montefiore Medical Center, Albert Einstein College of Medicine, Bronx, NY 10461, USA

**Keywords:** cardiomyocyte lipid metabolism drug targets, mitochondrial dysfunction therapeutics, iPSC-CM high-throughput screening, phospholipid transfer protein modulators, HDL function enhancers, cholesterol efflux pathway inhibitors, regenerative cardiology drug discovery, lipidomics-guided precision medicine, ABCA1/PLTP axis in cardiac therapy, novel metabolic modulators in heart disease

## Abstract

Phospholipid transfer protein (PLTP) is a lipid transfer protein classically studied in the context of plasma lipoprotein metabolism, high-density lipoprotein (HDL) remodeling, and cardiovascular disease risk. PLTP facilitates phospholipid transfer between lipoproteins and regulates HDL particle size and composition through interactions with apolipoprotein A-I and apolipoprotein A-II. While its systemic roles in cholesterol handling, reverse cholesterol transport, and inflammatory signaling are well established, the cell-autonomous functions of PLTP within cardiomyocytes remain poorly defined, particularly in human induced pluripotent stem cell-derived cardiomyocytes (iPSC-CMs). Extensive experimental and clinical studies demonstrate that PLTP enhances ABCA1-dependent cholesterol efflux primarily by stabilizing ABCA1 at the plasma membrane and by promoting the generation of lipid-poor apolipoprotein A-I and pre-β HDL particles, which serve as efficient cholesterol acceptors; the magnitude of these effects depends on cellular context, PLTP expression levels, and the availability of lipid acceptors. PLTP expression is metabolically regulated and widely distributed across tissues, including macrophages and other non-hepatic cells, supporting roles beyond circulating lipoprotein remodeling. Altered PLTP activity has been linked to atherosclerosis, cardiovascular disease, and inflammatory pathways, underscoring its relevance to cardiac pathophysiology. Emerging evidence further suggests that intracellular cholesterol distribution, rather than total cholesterol content alone, critically influences mitochondrial membrane composition, bioenergetics, and stress signaling in cardiomyocytes. These observations raise the possibility that PLTP-regulated lipid flux may indirectly shape mitochondrial function by modulating cellular cholesterol homeostasis. This review synthesizes current knowledge of PLTP biology, cholesterol metabolism, and lipoprotein remodeling, and integrates these concepts with emerging frameworks in cardiomyocyte lipid metabolism and mitochondrial physiology. We highlight human iPSC-derived cardiomyocytes as a strategic and translationally relevant platform to investigate PLTP’s non-canonical, cell-intrinsic roles, identify critical knowledge gaps, and propose future directions for elucidating how PLTP may influence mitochondrial function in human cardiac cells.

## 1. Introduction

### 1.1. Cholesterol Handling as a Determinant of Cardiomyocyte Function

Cholesterol homeostasis is a fundamental determinant of cardiomyocyte structure and function [1,2]. Beyond its role as a membrane constituent, cholesterol regulates membrane fluidity, receptor organization, and signal transduction, thereby influencing cardiomyocyte excitability and contractile performance [3,4]. Tight spatial control of cholesterol distribution is especially critical for organelle integrity, particularly within mitochondria, where membrane lipid composition governs electron transport chain organization, mitochondrial dynamics, and susceptibility to apoptotic signaling [5,6]. Emerging conceptual frameworks now emphasize cholesterol as a dynamically trafficked metabolite rather than a static membrane component, highlighting its active transport into mitochondria and its capacity to modulate mitochondrial bioenergetics—an axis to which cardiomyocytes appear unique sensitive given their high energetic demand [7,8].

At the systemic level, disruptions in cholesterol handling are highly prevalent. Epidemiological data from recent National Health and Nutrition Examination Survey analyses indicate that low levels of high-density lipoprotein cholesterol (HDL-C)—traditionally regarded as cardioprotective—affect approximately 13.8% of U.S. adults, with a marked sex disparity (≈21.5% in men versus ≈6.6% in women, 2021–2023) [9,10]. Reduced HDL availability compromises reverse cholesterol transport from peripheral tissues, including macrophage foam cells, thereby promoting cholesterol accumulation, vascular inflammation, and atherogenesis [11,12,13]. While these effects are classically framed within vascular pathology, they also raise important questions regarding how systemic lipid dysregulation intersects with cardiomyocyte-intrinsic cholesterol handling.

Because cardiomyocytes rely predominantly on oxidative phosphorylation (OXPHOS) to sustain continuous mechanical work, even modest perturbations in mitochondrial membrane lipid composition can impair respiratory efficiency, elevate reactive oxygen species production, and limit adaptive stress responses [14,15]. These properties render cardiomyocytes particularly vulnerable to disturbances in intracellular lipid trafficking, positioning cholesterol handling as a critical nexus linking systemic metabolic state to mitochondrial function and cellular resilience within the myocardium [2,16].

### 1.2. Overview of Phospholipid Transfer Protein (PLTP) Biology

Phospholipid transfer protein (PLTP) is a lipid transfer protein best characterized for its role in plasma lipoprotein metabolism [17,18]. Canonically, PLTP facilitates the transfer of phospholipids between lipoproteins and regulates high-density lipoprotein (HDL) particle size and composition [19,20]. Through interactions with apolipoprotein A-I (apoA-I) and apolipoprotein A-II (apoA-II), PLTP promotes HDL remodeling, generating pre-β HDL particles and fused HDL species that serve as efficient acceptors for cellular cholesterol efflux [21]. In plasma, PLTP exists in at least two functional forms: an active form predominantly associated with apoA-I–containing HDL particles and a low-activity form linked to apoE-containing lipoproteins [17,22]. These distinct pools underscore the functional heterogeneity of PLTP and suggest context-dependent biological effects [23].

### 1.3. Beyond Plasma: Tissue Expression and Metabolic Regulation of PLTP

Beyond its circulating lipoprotein-associated pool, several studies have reported intracellular PLTP localization, suggesting that PLTP may also function in cell-autonomous lipid handling. Consistent with this concept, intracellular PLTP has been detected in the endoplasmic reticulum, Golgi apparatus, and secretory vesicles of hepatocytes and macrophages, locations that are integral to lipid trafficking and lipoprotein assembly within the secretory pathway. In macrophages, intracellular PLTP expression has been shown to regulate cholesterol efflux capacity and inflammatory signaling independent of circulating HDL levels, indicating that locally expressed PLTP can influence cellular lipid homeostasis through autocrine or paracrine mechanisms. Together, these observations support the concept that PLTP exists in both extracellular and intracellular pools, potentially mediating distinct biological functions depending on its cellular context.

Although these intracellular functions have been characterized primarily in hepatocytes and macrophages, they provide a conceptual framework suggesting that cardiomyocyte-expressed PLTP may similarly regulate intracellular lipid trafficking and organelle membrane composition.

In parallel with its intracellular localization, PLTP expression is not restricted to the circulation but is observed across multiple tissues, including macrophages, lung, and placenta, supporting roles beyond systemic lipid transport [17,21]. Both PLTP expression and activity appear to be dynamically regulated by metabolic cues. High-fat diets and glucose availability increase PLTP levels, whereas insulin signaling suppresses its expression [18,24]. This metabolic responsiveness positions PLTP as a lipid regulator capable of integrating nutritional state with lipid handling at both tissue and cellular levels [16,25]. Importantly, PLTP activity has been linked to inflammatory signaling and cardiovascular disease risk, suggesting that its biological roles extend beyond lipoprotein remodeling to broader aspects of cardiometabolic homeostasis [23,26].

### 1.4. Rationale for iPSC-Derived Cardiomyocyte Models

Human induced pluripotent stem cell-derived cardiomyocytes (iPSC-CMs) [27,28,29,30,31,32,33,34,35,36,37,38,39,40,41] provide a human-relevant platform to interrogate cardiomyocyte-intrinsic metabolic pathways under controlled conditions [16].

However, it is important to note that iPSC-CMs do not fully recapitulate the structural, electrophysiological, and metabolic maturity of adult cardiomyocytes. Instead, these cells more closely resemble fetal-to-neonatal cardiomyocytes, particularly with respect to mitochondrial organization, metabolic substrate preference, and contractile maturation. Consequently, iPSC-CMs should be interpreted as a human developmental cardiomyocyte model rather than a direct substitute for mature adult cardiomyocytes.

These cells enable the dissection of lipid handling and mitochondrial function independent of systemic lipoprotein confounders [5,14]. Despite extensive knowledge of PLTP in plasma and peripheral tissues, no direct studies have yet defined PLTP’s role in mitochondrial function in human iPSC-derived cardiomyocytes. Addressing this gap is essential to understanding whether PLTP influences cardiomyocyte bioenergetics through cell-autonomous modulation of cholesterol distribution and mitochondrial integrity [17,24].

## 2. Methods

### 2.1. Review Design and Scope

This article was designed as a focused narrative review to synthesize and integrate current knowledge on phospholipid transfer protein (PLTP) biology, cholesterol handling, and mitochondrial function, with particular emphasis on cell-autonomous mechanisms in human induced pluripotent stem cell-derived cardiomyocytes (iPSC-CMs).

Rather than performing a formal systematic review or meta-analysis, the objective was to critically integrate mechanistic, translational, and conceptual studies spanning lipid metabolism, cardiomyocyte bioenergetics, and mitochondrial membrane biology, and to identify knowledge gaps relevant to human cardiac models.

The review prioritized mechanistic plausibility, biological coherence, and translational relevance, especially where established plasma-centric PLTP biology intersects with emerging frameworks in intracellular cholesterol trafficking and mitochondrial regulation.

### 2.2. Literature Search Strategy

A structured literature search was conducted using PubMed/MEDLINE, Web of Science, and Google Scholar, covering publications from January 2000 through December 2025, reflecting the historical emergence of PLTP biology and the more recent expansion of iPSC-CM and mitochondrial lipid research.

Search terms were applied individually and in combination using Boolean operators (“AND,” “OR”) to maximize sensitivity while maintaining conceptual relevance. Core keyword groups included:

PLTP and Lipid Biology

- “phospholipid transfer protein”;
- “PLTP cholesterol efflux”;
- “PLTP ABCA1”;
- “HDL remodeling PLTP”;
- “reverse cholesterol transport PLTP”.

Cardiomyocyte and Cardiac Metabolism

- “cardiomyocyte cholesterol metabolism”;
- “cardiac lipid homeostasis”;
- “mitochondrial cholesterol cardiomyocyte”;
- “cardiac bioenergetics lipid”.

Mitochondrial Membrane and Bioenergetics

- “mitochondrial membrane cholesterol”;
- “respiratory supercomplex lipid”;
- “mitochondrial lipid composition”;
- “cholesterol oxidative phosphorylation”.

Human iPSC-Derived Cardiomyocytes

- “iPSC-derived cardiomyocytes metabolism”;
- “iPSC-CM mitochondrial function”;
- “human cardiomyocyte lipid trafficking”;
- “cardiomyocyte maturation mitochondria”.

Reference lists of highly cited primary studies and authoritative reviews were manually screened to identify additional foundational or emerging articles not captured through database queries, particularly in mitochondrial lipid biology and intracellular cholesterol trafficking.

### 2.3. Study Selection and Inclusion Criteria

Articles were selected based on conceptual relevance rather than experimental uniformity, consistent with the narrative review format.

Inclusion criteria:

- Peer-reviewed original research or authoritative reviews;
- Studies addressing PLTP function, cholesterol trafficking, HDL remodeling, or ABCA1 regulation;
- Experimental or conceptual work linking lipid homeostasis to mitochondrial structure or bioenergetics;
- Studies involving cardiomyocytes, cardiac tissue, or human iPSC-derived cardiomyocytes;
- Foundational mechanistic studies from non-cardiac systems (e.g., macrophages, hepatocytes, neurons) when directly informative for intracellular cholesterol–mitochondria interactions.

Exclusion criteria:

- Studies limited to descriptive plasma lipid associations without mechanistic insight;
- Non-cardiac models lacking relevance to mitochondrial or intracellular lipid regulation;
- Abstract-only publications, non-peer-reviewed sources, or articles without accessible full text.

No restrictions were imposed based on species; however, human data were prioritized, followed by mammalian models with conserved lipid and mitochondrial pathways.

### 2.4. Data Extraction and Conceptual Synthesis

Rather than extracting quantitative outcome measures, selected studies were analyzed for:

- Primary biological mechanism (e.g., lipid transfer, transporter stabilization, mitochondrial membrane effects);
- Cellular context (plasma vs. cell-autonomous, organelle-specific effects);
- Relevance to cardiomyocyte metabolism or mitochondrial function;
- Experimental strength (genetic manipulation, functional assays, organelle-resolved analyses).

Findings were grouped thematically into:

1. Canonical PLTP functions in lipoprotein remodeling and cholesterol efflux;
2. Intracellular cholesterol trafficking and mitochondrial membrane biology;
3. Cardiomyocyte-specific metabolic vulnerabilities;
4. Translational relevance using human iPSC-derived cardiomyocyte models.

Conceptual figures and summary tables were constructed to distinguish established observations from hypothesis-generating interpretations, explicitly labeling proposed mechanisms where direct experimental evidence in cardiomyocytes is lacking.

### 2.5. Methodological Transparency and Limitations

This review does not claim exhaustive coverage of all PLTP-related literature and does not employ formal risk-of-bias scoring or meta-analytic techniques. Instead, it emphasizes mechanistic coherence and translational logic, acknowledging that many proposed PLTP–mitochondria links remain inferential.

Where extrapolations are made—from plasma biology, non-cardiac cells, or animal models—these are explicitly framed as conceptual extensions, not definitive conclusions. This approach is intended to provide a hypothesis-generating framework to guide future experimental studies in human cardiomyocytes.

An additional limitation is that human iPSC-derived cardiomyocytes represent developmentally immature cardiomyocytes, typically resembling fetal or neonatal stages rather than fully mature adult myocardium; therefore, mechanistic interpretations related to PLTP function should be considered within this developmental context.

### 2.6. Ethical Considerations

No new human or animal experiments were performed for this review. All referenced studies were conducted in accordance with their respective ethical standards.

## 3. PLTP in Cholesterol Efflux and Lipoprotein Remodeling

### 3.1. PLTP-Mediated HDL Remodeling

Phospholipid transfer protein (PLTP) functions pivotally in modulating high-density lipoprotein (HDL) structural dynamics through its capacity to transfer surface phospholipids between lipoprotein particles [17,18,19]. In plasma, PLTP promotes the generation of pre-β HDL, a nascent HDL subclass with high affinity for cholesterol efflux, alongside larger, fused HDL species arising from phospholipid enrichment and particle coalescence [20,21,22,23]. This remodeling process enhances the efficiency of HDL as a cholesterol acceptor by increasing particle surface area and apoA-I accessibility, thereby facilitating greater interaction with cellular cholesterol transporters [12,22,24]. The balance between pre-β HDL availability and fused HDL formation is a key determinant of HDL functional capacity, implicating PLTP as a principal regulator of HDL acceptor efficiency independent of HDL concentration alone [25,26].

### 3.2. Interaction Between PLTP and ABCA1

At the cellular interface, PLTP engages with ATP-binding cassette transporter A1 (ABCA1), a transmembrane lipid efflux pump critical for nascent HDL assembly [12,17]. Biochemical evidence indicates that PLTP can bind to and stabilize ABCA1 on the plasma membrane, reducing ubiquitin-mediated degradation and prolonging its functional lifetime [18,22,27]. This stabilization augments ABCA1-dependent cholesterol efflux, thereby facilitating the transfer of cellular cholesterol and phospholipids onto lipid-poor apolipoproteins [13,26,28]. While systemic PLTP activity influences plasma HDL profiles, the interaction with ABCA1 underscores a cell-autonomous efflux mechanism, wherein PLTP enhances local cholesterol export capacity irrespective of circulating lipoprotein levels [20,29]. Distinguishing these cellular effects from systemic lipoprotein remodeling is essential when considering tissue-specific lipid homeostasis [16,21].

### 3.3. Complexity of PLTP in Reverse Cholesterol Transport

Despite mechanistic links to cholesterol efflux, the role of PLTP in whole-organism reverse cholesterol transport (RCT) remains complex and context dependent [17,18]. Genetic models reveal that both PLTP deficiency and overexpression can diminish HDL mass without correspondingly altering macrophage RCT in vivo, indicating that HDL quantity is not a surrogate for functional cholesterol trafficking [12,22,30]. Similarly, altered PLTP expression may paradoxically elevate HDL functional markers while failing to enhance net cholesterol mobilization from peripheral tissues [20,21]. These observations emphasize that cholesterol flux through cellular efflux pathways can be decoupled from static measures of plasma HDL, and that the efficacy of lipid transport requires assessment of dynamic transporter interactions and acceptor particle functionality [13,26,31].

### 3.4. Implications for Cardiomyocyte Cholesterol Homeostasis

Collectively, PLTP-mediated remodeling of HDL and enhancement of ABCA1-dependent efflux establish a framework for intracellular cholesterol management that extends beyond vascular cells [12,17]. In cardiomyocytes, where plasma lipoprotein uptake is limited relative to macrophages and enterocytes, intracellular cholesterol handling—rather than systemic RCT—may be the critical axis governing lipid distribution and organelle lipid composition [4,16]. This conceptual shift prioritizes the investigation of PLTP’s cell-intrinsic actions in cardiomyocyte cholesterol trafficking and homeostasis, setting the stage for exploring its influence on mitochondrial function and metabolic integrity [1,6].

## 4. PLTP, Cardiovascular Disease, and Inflammation

### 4.1. PLTP Activity as a Cardiovascular Risk Marker

Phospholipid transfer protein (PLTP) activity has emerged as a quantitative biomarker linked to cardiovascular disease (CVD) risk in human populations [22,32,42]. Epidemiological analyses consistently show that elevated plasma PLTP activity correlates with adverse lipid profiles, higher triglyceride levels, and increased incidence of coronary artery disease, independent of traditional risk factors [17,18,33]. Genetic studies further indicate that low-activity PLTP phenotypes—resulting from natural variants that reduce protein expression or function—are associated with altered HDL subspecies distributions and modified lipid signatures, although their influence on clinical outcomes can be context dependent [20,34,43]. These genotype–phenotype associations underscore PLTP’s role in systemic lipid regulation and support its use as a risk stratifier in cardiometabolic profiling [21,29,35].

### 4.2. PLTP in Atherosclerosis Models

Preclinical models have been instrumental in dissecting PLTP’s contributions to atherogenesis [17,18,36]. Murine models with PLTP deficiency exhibit reduced HDL particle size and altered lipoprotein profiles, while PLTP overexpression can accelerate lipoprotein remodeling, increasing large HDL and intermediate lipoprotein fractions [20,22,37] (Table 1). Paradoxically, both deficiency and overexpression models have demonstrated attenuated atherosclerotic lesion development under certain dietary conditions, suggesting that PLTP’s impact on atherogenesis depends on the balance between lipoprotein remodeling and lipid transport efficiency [32,33,38]. These findings emphasize that PLTP’s modulation of very-low-density lipoprotein (VLDL) and HDL dynamics affects systemic lipid distribution and metabolic fluxes in ways that transcend simple measures of lipoprotein concentration, implicating complex regulatory networks in vascular lipid homeostasis [12,29].

This table summarizes how divergent genetic manipulation of PLTP alters HDL characteristics, systemic lipid handling, and atherosclerotic lesion development in preclinical models. Notably, both PLTP deficiency and overexpression can attenuate atherogenesis under specific dietary or metabolic contexts, highlighting a paradoxical and context-dependent role of PLTP in vascular lipid homeostasis. These findings underscore that PLTP influences atherosclerosis not merely through quantitative changes in HDL levels, but via integrated effects on lipoprotein remodeling, VLDL secretion, inflammatory signaling, and lipid transport efficiency across metabolic states.

### 4.3. Anti-Inflammatory and Oxidative Stress Modulation

Beyond lipid transport, PLTP influences inflammatory and oxidative pathways that intersect with CVD pathophysiology [18,22]. Experimental evidence suggests that PLTP activity can attenuate inflammasome activation, including NLRP3-dependent signaling, in macrophages and vascular cells, potentially limiting pro-inflammatory cytokine release and immune cell recruitment during lesion development; however, the magnitude and direction of this effect may vary depending on cellular lipid status and experimental context [26,39]. Additionally, in central nervous system models, PLTP affects tissue vitamin E distribution and cellular resistance to oxidative stress, providing a precedent for non-lipoprotein roles in redox regulation [26,41]. Collectively, these observations position PLTP as a mediator not only of lipid flux but also of inflammation and oxidative resilience—processes that are integrally linked to cardiac injury and remodeling [16,29].

### 4.4. Relevance to Cardiomyocyte Stress and Survival

Inflammation and dysregulated lipid metabolism synergize to compromise cardiomyocyte viability through mitochondrial dysfunction and impaired bioenergetics [1,16]. Accumulating lipid intermediates and pro-inflammatory signals disrupt mitochondrial respiratory chain efficiency, increase reactive oxygen species production, and promote apoptotic pathways [6,14]. In this context, PLTP’s capacity to influence lipid efflux, lipoprotein remodeling, and inflammatory signaling suggests it may act as a modifier of cardiomyocyte stress resilience [17,29]. Elucidating how PLTP mediates these intersecting pathways in human iPSC-derived cardiomyocytes could reveal novel mechanisms by which lipid inflammation interfaces with mitochondrial integrity and contractile performance [44,45,46].

## 5. Cholesterol–Mitochondria Interactions in Cardiomyocytes

### 5.1. Cholesterol in Mitochondrial Membranes

Mitochondrial membranes possess a distinct lipid milieu that is crucial for organelle function [14,15]. Cholesterol is a minor but functionally significant component of both the outer and inner mitochondrial membranes, where increased mitochondrial cholesterol content has been shown to reduce membrane fluidity, alter membrane packing, and disrupt the organization of respiratory chain complexes and associated membrane proteins [1,6,42,43,47,48,49,50,51,52]. Alterations in membrane cholesterol content can stiffen lipid bilayers [53,54,55,56], affecting the mobility [57,58,59] and functional coupling of electron transport chain (ETC) complexes [2,7,60]. Given that ETC efficiency underpins ATP synthesis, perturbations in cholesterol homeostasis may compromise oxidative phosphorylation by destabilizing respiratory supercomplex assembly and electron flow [1,61,62]. In addition, mitochondrial membrane cholesterol content can modulate apoptotic signaling pathways by influencing the activity of proteins such as Bax and Bak, which interact with cholesterol-rich microdomains to facilitate cytochrome c release [6,61,63,64]. Thus, intracellular cholesterol distribution affects both bioenergetic capacity and susceptibility to cell death pathways [16,65].

### 5.2. Intracellular Lipid Trafficking and Mitochondrial Function

Intracellular cholesterol does not distribute uniformly; distinct pools exist that are regulated independently of plasma lipoprotein levels [2,6]. Cardiomyocytes possess cholesterol pools associated with the plasma membrane, endolysosomal system, and mitochondrial membranes, with trafficking between these pools governed by vesicular and non-vesicular transport mechanisms [4,66,67]. HDL-independent cholesterol trafficking pathways contribute to intracellular homeostasis, with proteins such as NPC1/2 and oxysterol binding protein-related proteins facilitating cholesterol movement from late endosomes to mitochondria [1,68,69,70]. Mechanistic studies in macrophages and non-cardiac models have demonstrated that intracellular cholesterol trafficking to mitochondria is tightly regulated, with disruptions leading to altered electron transport chain organization, ROS generation, and impaired apoptotic signaling [8,71]. These insights support the hypothesis that similar pathways may operate in iPSC-derived cardiomyocytes, potentially intersecting with PLTP-mediated lipid flux.

Crucially, the distribution of cholesterol among organelles—rather than its total abundance—is a determinant of mitochondrial function [14,15]. Excess cholesterol in mitochondrial membranes has been shown to impair cardiolipin synthesis and destabilize inner membrane protein complexes, whereas cholesterol depletion can compromise membrane integrity and increase susceptibility to lipid peroxidation [1,6]. These findings highlight the importance of compartmentalized lipid regulation in maintaining organelle bioenergetics and redox homeostasis [16,46,72].

### 5.3. Conceptual Role of PLTP in Mitochondrial Lipid Homeostasis

**Figure 1:**
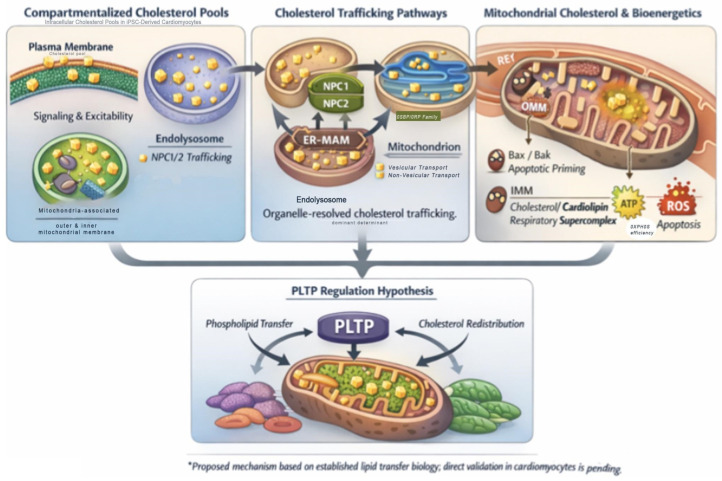
The Intracellular PLTP–Mitochondria Axis in iPSC-CMs (signature hypothesis figure). This schematic illustrates a hypothesis-generating framework integrating established principles of intracellular cholesterol trafficking and mitochondrial membrane biology. Cardiomyocytes harbor distinct cholesterol pools associated with the plasma membrane, endolysosomal system, and mitochondria. Cholesterol trafficking between these compartments is mediated by vesicular and non-vesicular pathways, including NPC1/2, oxysterol-binding protein-related proteins, and mitochondria-associated ER membranes. Within mitochondria, cholesterol—although a minor lipid component—critically influences membrane fluidity, cardiolipin organization, respiratory supercomplex stability, and stress-induced apoptotic signaling. The model proposes a non-canonical, cell-autonomous role for phospholipid transfer protein (PLTP) in modulating intracellular lipid flux, thereby indirectly shaping mitochondrial membrane composition and bioenergetic efficiency in human iPSC-derived cardiomyocytes. Balanced intracellular cholesterol trafficking preserves mitochondrial membrane fluidity and respiratory supercomplex organization, thereby sustaining oxidative phosphorylation and cardiac stress resilience. Dysregulation of this axis may predispose cardiomyocytes to energetic failure and apoptotic sensitivity. *This schematic represents a hypothesis-generating framework derived from established lipid transfer and mitochondrial membrane biology; direct experimental validation of PLTP–mitochondria interactions in cardiomyocytes remains to be established.

Although direct evidence linking PLTP to mitochondrial lipid regulation in cardiomyocytes is currently lacking, mechanistic principles from lipid biology suggest plausible pathways by which PLTP-mediated lipid flux could influence mitochondrial environments (Figure 1) [16,22]. PLTP’s known role in facilitating phospholipid and cholesterol transfer between lipoprotein particles positions it as a regulator of lipid acceptor availability and intracellular lipid distribution [12,17].

In cardiomyocytes, cell-autonomous PLTP expression may intersect with intracellular lipid trafficking systems to influence the redistribution of cholesterol and phospholipids among membrane compartments, including compartments functionally coupled to mitochondria [63,73]. Conceptual models of mitochondrial cholesterol transit—such as described by Simmen & Pellegrini (2025) [7]—together with canonical intracellular routing pathways outlined by Luo et al. (2020) [68], provide a mechanistic framework through which PLTP-dependent lipid flux could indirectly modulate mitochondrial membrane composition. Such redistribution may affect inner mitochondrial membrane lipid order, cardiolipin organization, and the stability of respiratory supercomplex assemblies.

By shaping the balance of lipid species available for membrane incorporation or efflux, PLTP may therefore exert secondary control over mitochondrial bioenergetic efficiency and respiratory complex stability, without directly localizing to mitochondria [14,16,17] (Table 2). This hypothesis-generating framework integrates known lipid transfer biology with emerging concepts in organelle-resolved lipid regulation and provides a rationale for experimental interrogation in human iPSC-derived cardiomyocytes.

This table summarizes selected lipid-handling proteins that influence mitochondrial membrane composition and function through distinct mechanisms. It positions PLTP alongside recognized mitochondrial lipid regulators, not isolated. PLTP is included as a proposed modulator of intracellular phospholipid and cholesterol flux with potential indirect effects on mitochondrial membrane dynamics. In contrast, PCSK9 and the translocator protein (TSPO) have established roles in regulating cholesterol accumulation and mitochondrial membrane composition, respectively, with downstream effects on mitochondrial permeability transition, steroidogenic pathways, and metabolic stress responses. Collectively, these modulators illustrate convergent yet mechanistically distinct routes by which lipid regulation interfaces with mitochondrial bioenergetics and cellular resilience.

This conceptual framework contextualizes PLTP within the broader network of mitochondrial lipid regulators and bioengineering approaches that govern cardiomyocyte bioenergetics (Table 3), positioning PLTP as a putative modulator of intracellular lipid flux with downstream implications for mitochondrial membrane organization and stress resilience in iPSC-derived cardiomyocytes [5,56]. At the molecular level, comparison with established mitochondrial lipid regulators such as PCSK9 and TSPO further delineates PLTP’s non-canonical, indirect mode of action on mitochondrial membranes, highlighting key mechanistic distinctions among lipid-handling pathways (Table 2).

This table contrasts phospholipid transfer protein (PLTP) with established regulators of mitochondrial lipid composition and function that influence cardiomyocyte bioenergetics, with a focus on evidence derived from human iPSC-derived cardiomyocyte (iPSC-CM) models where available. PLTP is presented as a putative intracellular modulator of mitochondrial membrane lipid environments, based on its established role in lipid transfer and HDL remodeling, rather than on direct mechanistic validation in cardiomyocytes. In contrast, PCSK9–TSPO signaling, statin-induced cholesterol synthesis inhibition, cardiolipin–cholesterol interactions, CRISPR-based extracellular vesicle (EV) delivery, and biomaterial-driven mechanotransduction represent pathways with more direct experimental links to mitochondrial respiration, membrane potential, fatty acid oxidation (FAO), or biogenic capacity. Clinical correlations highlight contexts in which mitochondrial lipid dysregulation contributes to cardiac remodeling, heart failure progression, drug-induced toxicity, or regenerative strategies. Collectively, the table situates PLTP within a broader landscape of mitochondrial lipid regulators, emphasizing its hypothesis-generating role and the need for organelle-resolved, cell-autonomous validation in human cardiomyocyte systems.

Table 3 intentionally separates hypothesis-generating integration from mechanistically established pathways, reinforcing the need for organelle-resolved, cell-autonomous validation of PLTP function in human cardiomyocyte systems. While the regulators summarized above represent endogenous or pharmacologic modulators of mitochondrial lipid homeostasis, emerging bioengineering approaches—including CRISPR-based genome editing, extracellular vesicle delivery systems, and biomaterial interfaces—provide complementary strategies to experimentally and therapeutically manipulate these pathways, which is elaborated in Section 6.4.

## 6. Human iPSC-Derived Cardiomyocytes as a Platform to Study PLTP

### 6.1. Advantages of iPSC-CMs for Lipid and Mitochondrial Studies

IPSC-CMs provide a uniquely versatile system for dissecting cell-intrinsic lipid and mitochondrial biology [16,41,82]. Unlike primary cardiomyocytes or non-human models, iPSC-CMs preserve the donor’s human genetic background, including polymorphisms that affect PLTP expression or function [48,80,83]. This feature enables interrogation of genotype-dependent lipid handling in a physiologically relevant context [58,80,84,85,86,87]. Moreover, iPSC-CMs can be cultured under controlled metabolic conditions, enabling precise manipulation of lipid availability, nutrient composition, and oxidative stress [46,86,88]. Such environmental control facilitates the isolation of PLTP-specific effects on intracellular cholesterol distribution and mitochondrial dynamics (Table 4) [5,7,89].

In parallel, mechanistic frameworks derived from non-cardiac systems establish mitochondrial cholesterol trafficking as a dynamic, regulatable process [7,8,90], supporting the use of iPSC-CMs to experimentally probe how PLTP-mediated lipid redistribution may influence mitochondrial structure and oxidative phosphorylation. Compatibility with live-cell imaging, high-resolution respirometry, and reactive oxygen species (ROS) measurement further supports quantitative assessment of mitochondrial function in response to altered PLTP activity or cholesterol trafficking [6,55,91].

**Table 4:** Strategic Advantages of Human iPSC-Derived Cardiomyocytes for Studying PLTP-Dependent Lipid and Mitochondrial Biology.

Feature	Advanced for Pltp Research	Technical Depth	Primary Lipid Mechanism	Mitochondrial Effect (Observed vs. Proposed)
Human genetic context [80,84,86]	Enables interrogation of PLTP function without species-specific lipoprotein differences that confound murine HDL biology	Patient-specific or CRISPR-edited iPSC lines allow PLTP gain/loss-of-function under identical nuclear background	Intracellular cholesterol redistribution rather than plasma HDL remodeling	Proposed: Alters mitochondrial cholesterol accessibility and membrane rigidity
Cell-autonomous lipid handling [17,73,92]	Separates cardiomyocyte-intrinsic PLTP effects from systemic lipoprotein transport	Lipoprotein-free or defined lipid culture systems	ABCA1-coupled phospholipid/cholesterol efflux and intracellular trafficking	Proposed: Direct modulation of mitochondrial membrane composition
Mitochondrial immaturity as a probe [14,81,93]	Baseline metabolic plasticity sensitizes detection of lipid-driven mitochondrial remodeling	Quantifiable shifts in OXPHOS, FAO, and glycolysis during maturation	Cholesterol-dependent regulation of membrane fluidity	Observed: Mitochondrial lipid composition strongly governs ETC efficiency
Direct relevance to cholesterol–mitochondria axis [1,5,61,94]	Human platform to extend findings from PCSK9–TSPO–mitochondrial cholesterol studies	High-resolution respirometry, EM, and lipidomics	Cholesterol import into mitochondria via non-canonical pathways	Observed: Cholesterol accumulation impairs respiratory supercomplex assembly
Compatibility with organelle-resolved lipidomics [6,60,95]	Allows direct testing of PLTP effects on mitochondrial vs. ER lipid pools	Subcellular fractionation + MS-based lipidomics	Phospholipid/cholesterol partitioning across membranes	Observed (non-CM): Mitochondrial cholesterol excess disrupts OXPHOS
Inflammation–metabolism coupling [16,40,96]	Enables linking PLTP-dependent lipid handling to cardiomyocyte inflammatory stress	Cytokine stimulation, inflammasome readouts, ROS assays	Lipid-driven modulation of innate immune signaling	Proposed: Cholesterol redistribution alters mitochondrial ROS thresholds
Disease-in-a-dish modeling [85,86,97]	Models metabolic cardiomyopathies where HDL-independent lipid stress dominates	Metabolic stress, lipid overload, hypoxia-reoxygenation	Dysregulated intracellular cholesterol homeostasis	Observed: Mitochondrial dysfunction precedes contractile failure
Translational alignment with regenerative cardiology [41,42,43,44,45,46,47,48,49,50,51,52,53,54,55,98]	Direct bridge between PLTP biology and therapeutic mitochondrial optimization	Integration with EV delivery, CRISPR editing, maturation strategies	Lipid remodeling as a determinant of mitochondrial fitness	Proposed: PLTP modulation enhances stress resilience and survival
Mitochondrial cholesterol/cardiolipin insights [76]	Provides mechanistic basis for membrane lipid influence on MPT and ROS	Mechanistic lipid-membrane studies applicable to iPSC biochemical modeling	Mitochondrial cholesterol and cardiolipin interactions	Observed: Cholesterol/cardiolipin in mitochondrial membranes disrupts cardiolipin-dependent membrane organization, o increases membrane rigidity, and impairs OXPHOS efficiency and bioenergetic performance
Statin effects on iPSC-CM mitochondria [75]	iPSC-Cm evidence linking cholesterol synthesis perturbation to mitochondrial dysfunction	Functional mitochondrial assays available in iPSC-CMs	Cholesterol synthesis inhibition and mitochondrial respiration	Observed: Statins reduce respiration and potential in iPSC-CMs

This table summarizes the key features of human iPSC-derived cardiomyocytes (iPSC-CMs) that make them uniquely suited for investigating phospholipid transfer protein (PLTP) function in intracellular lipid handling and mitochondrial regulation. Columns highlight: (i) Feature—specific aspects of the model that enable mechanistic interrogation; (ii) Advanced for PLTP Research—how each feature facilitates the study of PLTP-dependent lipid trafficking, compartmentalized cholesterol distribution, and bioenergetic modulation; (iii) Technical Depth—experimental approaches and readouts enabled by iPSC-CMs, including CRISPR-based gain/loss-of-function, organelle-resolved lipidomics, live-cell imaging, high-resolution respirometry, and metabolic flux analyses; and (iv) Primary Lipid/Mitochondrial Mechanism—observed or proposed effects on mitochondrial cholesterol accessibility, membrane composition, respiratory supercomplex stability, oxidative phosphorylation (OXPHOS), reactive oxygen species (ROS) thresholds, and stress resilience.

This framework illustrates the translational potential of iPSC-CMs to bridge PLTP biology with mitochondrial function, inflammation–metabolism coupling, disease-in-a-dish modeling, and regenerative cardiology strategies, providing a human-relevant platform for mechanistic and therapeutic exploration. Selected literature is cited to anchor conceptual mechanisms and provide methodological context, including canonical intracellular cholesterol routing [59] and the conceptual mitochondrial cholesterol transit model [7].

### 6.2. Experimental Axes Enabled by iPSC-CM Models

iPSC-CMs allow for conceptual exploration of multiple interrelated axes linking PLTP function to cardiomyocyte metabolism. First, modulation of PLTP expression through genetic overexpression or knockdown can reveal its cell-autonomous roles in lipid trafficking and organelle lipid composition [17,84,92]. Second, cholesterol distribution profiling—using fluorescent sterol analogs, mass spectrometry, or subcellular fractionation—can clarify how PLTP activity affects intracellular cholesterol pools, particularly in membranes associated with mitochondria and endolysosomes [5,73,95]. Third, mitochondrial parameters, including respiration, membrane potential, ROS production, and dynamic remodeling, can be evaluated under conditions of altered PLTP expression or lipid stress [1,14,91]. Collectively, these axes permit the dissection of mechanistic links between PLTP-mediated lipid flux, mitochondrial membrane composition, and bioenergetic efficiency [16,80,94].

### 6.3. Translational Implications

**Figure 2:**
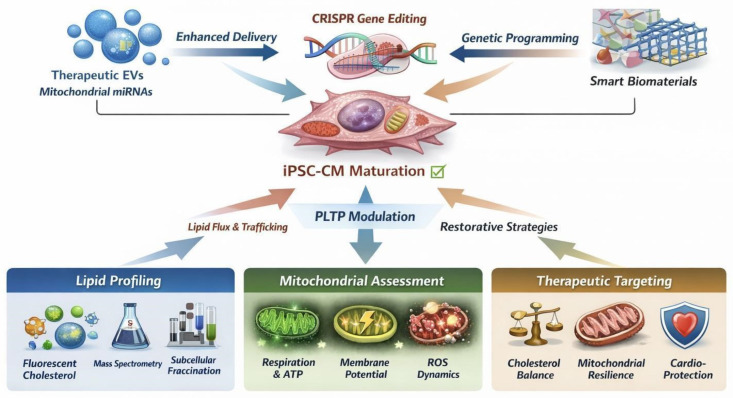
Multi-omic and Bioengineering Integration Roadmap for Next-Generation iPSC-CM Maturation and Regenerative Cardiology. This schematic illustrates a conceptual framework for integrating diverse experimental and bioengineering strategies to enhance the maturation, bioenergetic capacity, and translational utility of human iPSC-derived cardiomyocytes (iPSC-CMs). The diagram emphasizes relationships and synergy rather than detailed molecular mechanisms, highlighting how extracellular vesicles (EVs), CRISPR-guided mitochondrial biogenesis, biomaterial-mediated mechanotransduction, and PLTP-mediated lipid priming can converge to overcome maturation barriers and optimize mitochondrial function. The roadmap conveys how modulation of intracellular lipid flux—particularly via PLTP—may prime mitochondrial membranes for improved therapeutic responsiveness, including enhanced uptake of EV-encapsulated mitochondrial miRNAs, restoration of respiratory supercomplex stability, and resilience against oxidative stress. Conceptual arrows represent functional interactions and integrative strategies rather than proven causal pathways. This figure situates PLTP within a translationally relevant, multi-omic context, providing a human-relevant platform for mechanistic studies and regenerative cardiology applications in heart failure and metabolic cardiomyopathy models. The schematic emphasizes conceptual relationships rather than exhaustive molecular detail.

Insights derived from iPSC-CM studies have broad translational relevance [41,82,84]. In heart failure and metabolic cardiomyopathy, dysregulated lipid handling and mitochondrial dysfunction contribute to progressive contractile impairment [16,80,93]. Understanding PLTP’s modulatory role could identify novel strategies to restore intracellular cholesterol balance, enhance mitochondrial resilience, and prevent ROS-mediated damage (Figure 2) [28,31,94]. Additionally, iPSC-CMs provide a human-relevant platform to assess drug-induced mitochondrial toxicity, where perturbations in lipid trafficking may exacerbate mitochondrial vulnerability [34,35,36,37,38,39,40,41,44,45,46,47,48,74,91]. By linking PLTP function to cardiomyocyte bioenergetics, these models may guide therapeutic targeting in conditions characterized by compromised lipid and mitochondrial homeostasis, ultimately informing precision medicine approaches for cardiac disease [5,55,87].

### 6.4. Emerging Therapeutic and Bioengineering Strategies

Building upon the mechanistic insights and translational implications outlined above, a growing body of work has begun to explore interventional platforms that actively manipulate cardiomyocyte bioenergetics and maturation. Importantly, these approaches do not represent endogenous regulators of mitochondrial lipid homeostasis; rather, they function as engineered systems designed to modulate intracellular pathways, including those potentially influenced by PLTP-dependent lipid flux.

Among these, CRISPR-based mitochondrial engineering has emerged as a precise strategy to interrogate and enhance cardiomyocyte bioenergetics. In particular, EV-mediated delivery of CRISPR components (CRISPR-EVs) enables targeted gene editing within cardiomyocytes, facilitating controlled modulation of nuclear-encoded mitochondrial regulators [42,43,45,50,77,78,79]. Experimental studies indicate that such approaches can increase mitochondrial biogenesis, improve oxidative phosphorylation efficiency, and augment ATP production, thereby enhancing the energetic capacity of iPSC-derived cardiomyocytes. These platforms provide a scalable framework for regenerative therapy by coupling genetic precision with physiologically relevant delivery systems.

In parallel, EV-mediated delivery systems independent of genome editing have been increasingly utilized to transfer bioactive cargo—including mitochondrial proteins, RNAs, and metabolites—into recipient cardiomyocytes. These vesicles can influence mitochondrial dynamics, redox balance, and metabolic signaling, offering a non-genomic route to modulate cardiomyocyte function. Within the context of PLTP biology, EV-based strategies may be particularly relevant, as alterations in lipid composition and membrane organization could influence vesicle uptake, intracellular trafficking, and organelle targeting.

Complementing these molecular approaches, biomaterial-driven mechanotransduction platforms provide a biophysical means of directing cardiomyocyte maturation and metabolic remodeling [51,80,81]. Engineered substrates with defined stiffness, topography, and electrical conductivity have been shown to promote structural organization, sarcomere alignment, and a metabolic shift toward fatty acid oxidation (FAO)—hallmarks of mature cardiomyocytes. These changes are closely linked to mitochondrial development, including increased respiratory capacity and optimized membrane architecture. As such, biomaterials serve as critical tools in tissue engineering, enabling the recapitulation of physiologic mechanical cues that are otherwise absent in conventional in vitro systems.

Collectively, these bioengineering strategies—encompassing CRISPR-based gene modulation, EV-mediated cargo delivery, and biomaterial-induced mechanotransduction—offer complementary avenues to intervene in cardiomyocyte bioenergetics at genetic, molecular, and biophysical levels. These strategies do not represent endogenous mitochondrial lipid regulators but rather engineered approaches that can be leveraged to manipulate cardiomyocyte bioenergetics and maturation. When integrated with iPSC-CM platforms, they provide a powerful toolkit to experimentally test and therapeutically exploit mechanisms of intracellular lipid handling and mitochondrial function, including those hypothesized to involve PLTP.

Despite these advantages, iPSC-CM also presents important limitations. These cells typically exhibit an immature phenotype resembling fetal or neonatal cardiomyocytes, including reduced mitochondrial density, altered metabolic substrate preference, and incomplete electrophysiological maturation. Protein expression profiles and lipid metabolic pathways may therefore differ from those of fully mature adult cardiomyocytes. Consequently, findings obtained in iPSC-CM models should be interpreted within this developmental context and ideally complemented by validation in adult cardiomyocyte or in vivo systems.

## 7. Knowledge Gaps and Future Directions

Despite extensive characterization of PLTP in plasma lipid metabolism and HDL remodeling, direct evidence linking PLTP to mitochondrial function in human cardiomyocytes remains absent [16,17,22]. Most current mechanistic inferences are extrapolated from plasma or non-cardiomyocyte cellular models, leaving a critical gap in understanding whether PLTP influences mitochondrial bioenergetics through cell-autonomous regulation of intracellular cholesterol and phospholipid distribution [18,22,29]. Clarifying this relationship is essential, as cardiomyocyte mitochondria are highly sensitive to membrane lipid composition, which governs respiratory chain efficiency, supercomplex stability, and apoptotic susceptibility [14,61,94].

A primary knowledge gap concerns the separation of cell-autonomous effects from systemic influences [1,16,39]. In vivo, PLTP exerts both plasma-mediated lipoprotein remodeling and tissue-intrinsic functions [20,21]. Disentangling these axes in cardiomyocytes requires experimental systems—such as iPSC-CM—in which PLTP expression or enzymatic activity can be selectively increased or suppressed independently of circulating HDL or apoA-I levels, as supported by studies showing that cellular PLTP overexpression alters nascent HDL formation and promotes ABCA1-mediated cholesterol efflux in the absence of plasma lipoproteins [41,76,99]. Such approaches are needed to determine whether intracellular PLTP directly facilitates redistribution of cellular cholesterol toward mitochondrial membranes through phospholipid transfer and HDL remodeling pathways, or whether changes in PLTP activity influence mitochondrial membrane lipid composition indirectly by modifying extracellular lipid acceptor availability and phospholipid flux, as indicated by PLTP’s role in HDL particle remodeling and systemic HDL composition changes [7,73,90].

Another critical gap is the lack of organelle-resolved lipidomics in the context of PLTP function [6,15]. Traditional lipid profiling of whole-cell lysates cannot capture compartment-specific effects on mitochondrial, endolysosomal, or plasma membrane lipid pools [60,63]. Advanced mass spectrometry-based lipidomics and fluorescence sterol mapping can delineate how PLTP alters the abundance, distribution, and saturation state of cholesterol, phosphatidylcholine, and cardiolipin within mitochondria [68,69,95]. Such resolution is necessary to determine whether PLTP-mediated lipid flux can stabilize inner membrane protein complexes or preserve mitochondrial dynamics under stress [2,5,94].

Finally, integration with inflammatory and redox signaling remains largely unexplored [39,40,100,101]. Studies suggest that PLTP expression correlates with inflammatory markers in cardiovascular disease and may influence inflammatory signaling, whereas direct evidence that PLTP enhances NLRP3 inflammasome activation is lacking; in fact, PLTP has been reported to inhibit NLRP3 inflammasome signaling in septic cardiac models [102,103]. In macrophages and vascular cells, PLTP has been shown to affect NLRP3 inflammasome activity and oxidative stress, yet its role in regulating cardiomyocyte mitochondrial ROS production, cytokine signaling, and stress-induced apoptosis has not been systematically investigated [30,59,80,104]. Mapping these pathways in a cell-autonomous context will be essential for defining whether PLTP functions as a modulator of cardiomyocyte resilience under metabolic or pharmacological stress [4,16,57,80,96,105].

Overall, the current conceptual framework is hypothesis-generating, offering a foundation for future studies rather than definitive mechanistic conclusions [48,84]. Investigations that combine genetic manipulation of PLTP, organelle-resolved lipidomics, and functional assays of mitochondrial respiration and ROS in human iPSC-CMs will be crucial to validate these hypotheses [48,85,95]. Ultimately, such studies may uncover novel cardioprotective mechanisms, positioning PLTP not only as a regulator of lipid flux but also as a potential therapeutic target for conditions characterized by mitochondrial dysfunction, metabolic cardiomyopathy, or inflammation-driven cardiac injury [3,54,98].

## 8. Conclusions

PLTP is a well-established regulator of cholesterol handling, mediating HDL remodeling, pre-β HDL formation, and ABCA1-dependent cholesterol efflux [17,20]. These canonical roles underscore its importance in systemic lipid homeostasis and cardiovascular biology, linking PLTP activity to HDL functionality, inflammatory modulation, and cardiovascular disease risk [12,22]. Beyond these systemic effects, accumulating evidence suggests that PLTP’s influence may extend into cell-autonomous regulation of intracellular lipid pools, with potential consequences for organelle integrity and cardiomyocyte bioenergetics [5,16].

A conceptual expansion of PLTP biology highlights its putative involvement in mitochondrial function [6,45]. Mitochondrial membranes possess a distinct lipid composition, with cholesterol and phospholipids critically modulating membrane fluidity, electron transport chain supercomplex stability, and apoptotic signaling [14,61]. Intracellular cholesterol trafficking to mitochondria is tightly regulated by vesicular and non-vesicular pathways, and perturbations in this balance can impair oxidative phosphorylation, increase reactive oxygen species production, and compromise cardiomyocyte resilience under metabolic or pharmacological stress [2,73]. By facilitating phospholipid and cholesterol transfer, PLTP may indirectly shape mitochondrial membrane lipid environments, influencing respiratory efficiency, ROS generation, and stress adaptation. These hypotheses, while mechanistically plausible, remain largely untested in cardiomyocytes [7,15].

iPSC-CMs represent the missing experimental link to interrogate these cell-intrinsic functions [34,63]. iPSC-CMs preserve human genetic background, allowing for the study of genotype-dependent PLTP expression and functional variability, while providing controlled metabolic and pharmacological contexts [49,76]. Through organelle-resolved lipidomics, live-cell imaging, and mitochondrial functional assays, iPSC-CMs offer a platform to define how PLTP modulates intracellular cholesterol distribution, mitochondrial dynamics, and bioenergetic efficiency independently of systemic lipoprotein influences [83,84]. This system bridges the gap between established plasma-centric PLTP biology and emerging hypotheses regarding mitochondrial regulation, providing a path toward mechanistic clarity [46,50].

In conclusion, while PLTP is classically recognized for its roles in HDL remodeling and cholesterol efflux, its potential contributions to mitochondrial function in human cardiomyocytes represent a novel frontier [43,61]. Elucidating these cell-autonomous mechanisms may uncover previously unrecognized regulatory layers of cardiac metabolism, linking lipid handling to bioenergetic resilience and stress adaptation [54,77]. Understanding PLTP beyond plasma lipoproteins may ultimately reveal new therapeutic targets for heart failure, metabolic cardiomyopathy, and inflammation-driven cardiac injury, highlighting its central role in the integration of lipid biology, mitochondrial health, and cardiomyocyte survival [16,55].

## Figures and Tables

**Table 1:** Comparison of PLTP Effects in Murine Models.

Model Type	Primary Effect on HDL	Key Impact on Atherogenesis	Primary Mechanism
PLTP Deficiency [20,22]	Reduced size/Concentration	often attenuated (Paradoxical)	Reduced VLDL secretion, altered inflammatory response
PLTP Deficiency—metabolic/inflammatory context added [39]	Altered lipid profile & inflammation	Enhanced metabolic dysfunctin & pro-inflammatory state	Altered inflammatory response and lipid transport
PLTP overexpression [39,40]	Increased large HDL Fractions	Variable/attenuated	Enhanced remodeling; increased lipid transport efficiency

**Table 2:** Biological Modulators of Mitochondrial Lipid Homeostasis and Bioenergetics in iPSC-Derived Cardiomyocytes.

Regulator	Primary Lipid Mechanism	Mitochondrial Effect (Observed vs. Proposed)	Relevance to iPSC-CM Metabolism
PLTP [7,8,15,22,68]	Facilitates phospholipid and cholesterol transfer between lipoprotein particles; regulates HDL remodeling and cellular cholesterol efflux	Proposed: By altering intracellular lipid distribution, PLTP may influence mitochondrial membrane lipid composition, potentially affecting respiratory complex organization and ROS production. Actual direct effects in cardiomyocytes remain untested.	Intersection of lipid handling and mitochondrial function in iPSC-CMs; hypothesized regulator of organelle lipid homeostasis and bioenergetics.
PCSK9 [1,5]	Proprotein convertase that regulates LDL receptor (LDL-R) levels and indirectly influences cholesterol trafficking; promotes mitochondrial cholesterol accumulation by inducing TSPO degradation (thereby reducing TSPO-mediated cholesterol export from mitochondria)	Observed: Cardiomyocyte-specific PCSK9 deficiency leads to mitochondrial cholesterol accumulation, impaired electron transport chain activity, altered mitochondrial morphology, and reduced oxidative phosphorylation.	Directly links cholesterol regulation to mitochondrial bioenergetics in cardiomyocytes; relevant model for studying lipid-dependent metabolic dysfunction in iPSC-CMs.
TSPO [1,74,75,76]	Mitochondrial translocator protein involved in cholesterol transport into and within mitochondria; interacts with regulatory proteins.	Observed (cardiac context): Upregulated when PCSK9 is deficient and linked to mitochondrial cholesterol accumulation. Observed (animal models): Interacts with mitochondrial lipid import and may influence mitochondrial function, including oxidative phosphorylation and ROS production. Upregulates import of cholesterol across the outer mitochondrial membrane (cytosol) into the inner mitochondrial compartment therefore modulates mitochondrial membrane composition; upregulate cholesterol use for steroid/bile acid production, bile acid/steroidgenetic control; alters MPT threshold (sensitize the mPTP, effectively downregulating mitochondrial resistance to permeability transition)	TSPO is a mechanistic node for cholesterol import into mitochondria; its modulation affects mitochondrial lipid load and function—conceptually relevant to iPSC-CM modeling of lipid-induced mitochondrial dysfunction.
Statin (simvastatin, atorvastastin) [75]	Inhibits cholesterol synthesis	Observed: reduced basal and maximal respiration, decreased mitochondrial membrane potential in iPSC-CMs, mitochondrial dysfunction and intracellular acidification	Demonstrates how cholesterol synthesis perturbation alters mitochondrial bioenergetics in iPSC-CM

**Table 3:** Comparative Roles of PLTP and Established Mitochondrial Lipid Regulators in Cardiomyocyte Bioenergetics.

Regulator	Primary Cardiac Mechanism	Impact on iPSC-CM Bioenergetics	Clinical Correlation
PLTP [17]	Intercellular Lipid Flux & HDL Remodeling	putative influence on mitochondrial membrane lipid environment with downstream implications for ETC organization	Heart failure & HDL-C dysfunction
PCSK9 [7]	TSPO Degradation	Mitochondrial cholesterol accumulation; impaired oxphos	Post-MI Remodeling
CRISPR-EVs [50,77,78,79]	Targeted gene editing	Enhancement of mitochondrial biogenesis and ATP production	Regenerative therapy
Biomaterials [53,80,81]	Mechanotransduction	Structural maturity and fao shift	Tissue Engineering
Statin-induced mitochondrial impairment in iPSC-CMs [75]	Cholesterol synthesis inhibition	Reduced respiration and membrane potential, intracellular acification in iPSC-CMs	Implications for clinical statin mitochondrial side effects
Mitochondrial cholesterol/cardiolipin interaction [6,14,61,76]	Membrane permeabilization modulation by cardiolipin redox state	Cholesterol & peroxidized cardiolipin impacts membrane permeabilization and ETC function	Broad metabolic/membrane regulation relevance

## Data Availability

No new data were created or analyzed in this study. Data sharing is not applicable to this article.

## Abbreviations

HDL-C	High-Density Lipoprotein Cholesterol
PLTP	Phospholipid Transfer Protein
apoA-I	Apolipoprotein A-I
apoA-II	Apolipoprotein A-II
apoE	Apolipoprotein E
iPSC-CM	Induced Pluripotent Stem Cell-Derived Cardiomyocyte
OXPHOS	Oxidative Phosphorylation
ROS	Reactive Oxygen Species
ABCA1	ATP-Binding Cassette Transporter A1
RCT	Reverse Cholesterol Transport
CVD	Cardiovascular Disease
VLDL	Very-Low-Density Lipoprotein
NLRP3	NOD-, LRR- and Pyrin Domain-Containing Protein 3 (inflammasome)
ETC	Electron Transport Chain
NPC1/2	Niemann-Pick Type C1/2 proteins
TSPO	Translocator Protein (Mitochondrial)
FAO	Fatty Acid Oxidation
MPT	Mitochondrial Permeability Transition
